# Comparative analysis of virulence gene profiles of Escherichia coli from human and non-human sources in Rivers State, Nigeria

**DOI:** 10.1099/acmi.0.000776.v6

**Published:** 2024-07-25

**Authors:** Barira Azeez Abeni, Nnenna Frank-Peterside, Kome Otokunefor

**Affiliations:** 1 Department of Microbiology, Faculty of Science, University of Port Harcourt, Port Harcourt, Nigeria

**Keywords:** *Escherichia coli*, Nigeria, virulence profile

## Abstract

Traditionally, the presence of virulence features has been thought to be a key factor in differentiating pathogenic from commensal strains. An understanding of the virulence potential of *Escherichia coli* isolates from various sources is essential to shed light on potential contamination/transmission rates between the various sources. This study was therefore aimed at exploring the occurrence of specific virulence genes and gene profiles associated with *E. coli* from human and non-human sources in Rivers State, Nigeria. Two hundred samples from human (urine and faeces) and non-human (soil and poultry droppings) sources (50 each) were analysed using standard microbiological procedures. DNA was extracted from isolates presumptively identified as *E. coli* using the Presto Mini gDNA Bacteria-Kit Quick protocol following the manufacturer’s instructions. Isolate identities were confirmed using *E. coli*-specific 16S rRNA primers, and confirmed isolates were screened for the presence of six virulence genes [afimbriae binding adhesin (*afa*), type 1 fimbriae (*fimH*) and P-fimbrial usher protein (*papC*)], iron acquisition systems: *aer*obactin (*aer*), cytotoxic necrotizing factor I (*cnf1*) and alpha-hemolysin (*hly*). Results showed that all isolates harboured at least one of the tested virulence genes, with *fimH* (97%) as the most prevalent virulence gene and *papC* the least commonly occurring (35%). A higher occurrence of virulence genes was noted in non-human isolates, though *hly* and *cnf* were not detected at all in any of the isolates studied (0%). Ten different profiles were observed with the *afaCc-aer-fimH* profile the most commonly occurring virulence gene profile being in general (33.3%). For non-human isolates, however, *aer-afaCc-fimH-papC* was the most commonly occurring profile (42.9%). This study shows that the test *E. coli* from human and non-human sources do not carry distinct virulence gene profiles. Studies on a larger subset of isolates would however be necessary to determine if the virulence genes tested in this study really cannot be used to tell whether an isolate is from a human source or not in the South–South of Nigeria.

## Data Summary

All data associated with this work is reported within the article.

## Introduction

*Escherichia coli* largely exists as commensal bacteria present as normal flora in humans, animals and pristine soil. However, it can also present as a pathogen capable of causing a number of diseases such as urinary tract infections (UTIs), septicaemia, meningitis, wound infections and pneumonia [1]. These pathogenic strains have often been widely classified and found to be associated with a number of acquired virulence features. These virulence features confer pathogenic potential on their host organisms by equipping the strains with specific characteristics needed for causing disease. Traditionally, the presence of virulence features has been thought to be a key factor in differentiating pathogenic strains from commensals [2]. Actually, in some cases, pathogenic strains are thought to develop following the uptake of a virulence determinant by a harmless commensal [3].

These virulence determinants are numerous and necessary in order for pathogenic organisms to invade and evade host tissues and immunity, respectively. They include α-hemolysin, which facilitates the invasion of host tissues, and afimbrial adhesins (*afa*) which aid in the attachment and dissemination of pathogenic organisms to host cells and lead to persistence and recurrent infections. And *cnf1*, which is responsible for polymorphonuclear phagocytosis, brings about scarring of the epithelia of the bladder [4]. Additionally, there is the *papC* gene that is essential for the synthesis of specific fimbriae associated with pyelonephritis-associated pili (pap) type and the iron acquisition systems (*aer*obactin and yersiniabactin), which confer on bacteria the ability to scavenge iron when it is in deficit or in short supply [5].

More recently, some studies have found that ‘commensals’ from environmental samples also harbour some of these virulence determinants as well [6,7]. In some cases, this has been thought to be linked directly or indirectly to human activities [6,8]. Due to human and animal activity, it is possible that pathogenic strains of *E. coli* find their way into various non-clinical environments, especially via run-off. Such environmental contamination is thought to occur more commonly in low- and middle-income countries due to poorer sanitary conditions [9].

Considering the increasing need to apply One Health approach to understanding and curbing infectious diseases and improving the quality of human life, an understanding of the virulence potential of *E. coli* isolates from various sources is essential. This information would shed light on potential contamination/transmission rates between the various sources, indicating where control measures are needed.

Quite a number of studies in the south-west of Nigeria have actually focused on assessing the occurrence of several virulence genes in *E. coli* from various sources [10,11], with a number focused on isolates from environmental sources [12,14]. This information from the South–South of Nigeria, where Rivers State is found, is lacking, with most reports coming out of our laboratory [15,16], and the comparative angle is missing. This study is therefore aimed at assessing the preponderance of specific virulence genes associated with *E. coli* from various sources in a bid to analyse variations in virulence gene profiles of *E. coli* from human and non-human sources in Port Harcourt, Rivers State.

## Methods

### Study details

This study was conducted at the Microbiology Laboratory, University of Port Harcourt and the Molecular Biology Laboratory, African Biosciences in Ibadan, Oyo State, Nigeria. The study involved the use of urine and faecal samples from the University of Port Harcourt Teaching Hospital and soil and poultry droppings over a 4-month period. All samples were collected and tested immediately over time without storage. A total of two hundred samples (50 from each source) were collected and analysed. Using standard microbiological procedures, all samples were aseptically cultured and isolates were identified therein. In summary, samples showing characteristic *E. coli* colonies (green metallic sheen) on eosin methylene blue agar (HiMedia, India) were purified and their identities were confirmed biochemically using conventional biochemical tests including oxidase, indole, methyl-red, Voges–Proskauer, citrate, starch hydrolysis, sugar fermentation, urease, motility, catalase and triple sugar iron fermentation tests [17].

### DNA extraction

The genomic DNA of *E. coli* isolates was extracted using Presto Mini gDNA Bacteria-Kit Quick in accordance with the manufacturer’s directions. The quantity and size of DNA were then determined using gel electrophoresis (100 V for 30 min) and the 1 kb GeneRuler DNA Ladder (Thermo Fisher Scientific).

### Molecular confirmation of *E. coli* isolates

*E. coli* isolates were identified molecularly via the use of *E. coli*-specific 16S rRNA gene fragments of Ec16 primers (F 5′-GACCTCGGTTAGTTCACAGA-3′ and R 5′-CACACGCTGACGCTGACCA-3′) as previously described [18].

### Molecular detection of *E. coli* virulence genes

The presence of virulence genes among *E. coli* isolates was screened by PCR targeting adhesins: *afa*) [19], type 1 fimbriae (*fimH*) [20], P-fimbrial usher protein (*papC*) [19]; iron acquisition systems: *aer*obactin (*aer*) [20]; toxins: cytotoxic necrotizing factor I (*cnf1*) [21] and alpha-hemolysin (*hly*) [20], as previously described in the reference studies.

In brief, PCR amplifications were carried out in a DNA thermocycler. The cycling conditions involved an initial denaturation at 95 °C for 3 min. This was followed by 35 cycles of denaturation at 95 °C for 30 s, primer-specific annealing temperatures (Table 1) for 30 s and extension at 72 °C for 40 s, then a final extension at 72 °C for 10 min. Amplification products were then visualized following separation using agarose gel electrophoresis stained with ethidium bromide under a gel-doc/UV transilluminator [22].

**Table 1.** Outline of virulence factor, target gene and primers

Virulence factor	Target gene(s)	Primer sequence (5′–3′)	Size of amplicon (bp)	Annealing temp. (°C)	References
Type 1 fimbriae	*fimH*	F: AACAGCGATGATTTCCAGTTTGTGTG	465	65	[20]
		R: ATTGCGTACCAGCATTAGCAATGTCC			
Afa adhesins	*afaCc*	F: CGGCTTTTCTGCTGAACTGGCAGGC	672	64	[19]
		R: CCGTCAGCCCCCACGGCAGACC			
Hemolysin	*hly*CA region	F: AGATTCTTGGGCATGTATCCT	556	65	[20]
		R: TTGCTTTGCAGACTGTAGTGT			
Cytotoxic necrotizing factor	*cnf*	TTATATAGTCGTCAAGATGGA	693	58	[21]
		CACTAAGCTTTACAATATTGA			
P fimbriae	*pap*C	F: GACGGCTGTACTGCAGGGTGTGGCGR: ATATCCTTTCTGCAGGGATGCAATA	328	65	[19]
*Aer*obactin	*aer*	F: AAACCTGGCTTACGCAACTGTR: ACCCGTCTGCAAATCATGGAT	269	60	[21]

### Statistical analysis

SPSS 17 statistical software (SPSS Inc., Chicago, IL, USA) was used for the statistical analysis. Pearson chi-square (X^2^) test was employed to compare the percentages obtained for genotypic characteristics and virulence genes. *P*-value was considered significant when it was less than 0.05.

**Chi-square test statistic:** It is defined as

$$\chi^{2}=\sum_{i=1}^{n}\frac{(O_{i}-E_{i}{)}^{2}}{E_{i}}$$

Where *O_i_* is the observed number of cases in category i, and *E_i_* is the expected number of cases in category i.

## Results

From the 200 samples collected in total, 98 isolates were presumptively identified as *E. coli* using standard biochemical tests. Only 60 of these were however molecularly confirmed to be *E. coli* based on the presence of the *E. coli*-specific 16S rRNA fragment (Table 2).

**Table 2.** Molecular distribution of confirmed *E. coli* isolates by source in the study

Specimen	No. sampled	Phenotypically identified as*E. coli* (%)	No. molecularly identified to be*E. coli* (%)
Urine	50	31 (62)	23 (74.2)
Faeces	50	31 (62)	16 (51.7)
Soil	50	10 (20)	4 (40)
Poultry droppings	50	26 (52)	17 (65.4)
Total	200	98 (49)	60 (61.2)

An assessment for the presence of the select six test virulence genes revealed that all isolates harboured at least one of the tested virulence genes giving a 100% occurrence of virulence genes in the test isolates. A wide variation in the specific occurrence of the virulence genes however existed (Table 3), with *fimH* (97%) occurring as the most prevalent virulence gene among all the isolates and *papC* the least commonly occurring (35%). *Hly* and *cnf* were not detected at all in any of the isolates studied (0%). Source variation existed in the occurrence of these test virulence genes (Table 3), with a higher occurrence of virulence genes noted in isolates from non-human sources. As a whole, isolates from human faeces had a lower occurrence of the test virulence genes, though there was no significant statistical difference (Table 4).

**Table 3.** Source-based variation in the occurrence rate of test virulence genes

Virulence genes	No. (*n* = 60)(% occurrence)	Human (*n* = 39)*N* (%)	Non-human (*n* = 21)*N* (%)	*P*-value
***fimH***	58 (96.7)	37 (97)	21 (100)	0.291
***aer***	46 (76.75)	27 (69)	19 (95.2)	0.048
***hly***	0 (0)	0 (0)	0 (0)	
***afa***	37 (61.7)	20 (51.3)	17 (81)	0.024
***pap***	21 (35)	11 (28.2)	10 (47.6)	0.133
***cnf***	0 (0 %)	0 (0)	0 (0)	

**Table 4.** Variation in the occurrence of virulence genes based on a specific source

Virulence genes	Urine (*n* = 23)*N* (%)	Faeces (*n* = 16)*N* (%)	Soil (*n* = 4)*N* (%)	Poultry droppings(*n* = 17) *N* (%)	*P*-value
***fimH***	23 (100)	14 (87.5)	4 (100)	17 (100)	0.128
***aer***	17 (73.9)	10 (62.5)	4 (100)	15 (88.2)	0.223
***hly***	0 (0)	0 (0)	0 (0)	0 (0)	
***afa***	14 (60.8)	6 (37.5)	3 (75)	14 (82.4)	0.062
***pap***	8 (34.8)	3 (18.8)	3 (25)	9 (52.9)	0.218
***cnf***	0	0 (0)	0 (0)	0 (0)	

**Fig. 1.**
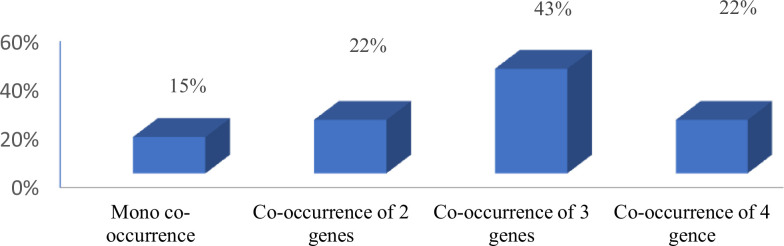
Co-occurrence of virulence genes in test *E. coli* isolates.

**Fig. 2.**
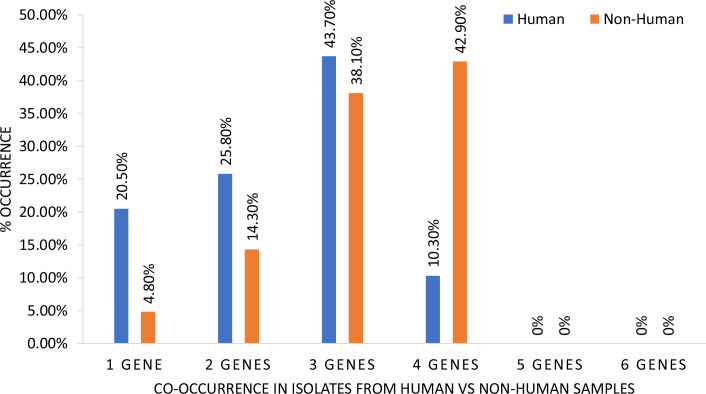
Source-based effect on co-occurrence of virulence genes.

An assessment of the co-occurrence of the test virulence genes showed that the majority of isolates (43%) exhibited a co-occurrence of three of the test genes (Fig. 1). Analysing co-occurrence based on sample source showed that in the non-human isolates, the more commonly occurring phenomenon (observed in 42.9% of isolates) was the co-occurrence of 4 virulence genes (Fig. 2).

Based on the combination of virulence genes detected, virulence gene profiles were generated for each isolate and 10 different profiles were observed (Table 5). In general, the *afaCc-aer-fimH* was the most commonly occurring virulence gene profile, though specifically for non-human isolates, the *aer-afaCc-fimH-papC* was the most commonly occurring profile. Four virulence gene profiles were present in the human isolates but absent in the non-human isolates, while one of the profiles was present among the non-human isolates but absent from the human isolates. A combination of *fimH* and *aer* genes was observed in 64.1% of human isolates and 95.2% of non-human isolates.

**Table 5.** Virulence gene profiles and association with specific test isolates

**S/No**	**Virulence profiles**	**No.(% occurrence)**	**Human(n=39)N(%)**	**Non-human (n=21)N(%)**	***P*-value**
1	*fimH*	9 (15)	8 (20.5)	1 (4.8)	0.033
2	*aer-afaCc*	1 (1.7)	1 (2.6)	0 (0)	0.5
3	*aer-fimH*	8 (13.3)	6 (15.4)	2 (9.5)	0.038
4	*aer-papC*	1 (1.7)	1 (2.6)	0 (0)	0.5
5	*afaCc-fimH*	1 (1.7)	0 (0)	1 (4.8)	0.5
6	*fimH-papC*	2 (3.3)	2 (5.1)	0 (0)	0.5
7	*aer-afaCc-fimH*	20 (33.3)	13 (33.3)	8 (33.3)	0.5
8	*aer-fimH-papC*	3 (5)	2 (5.2)	1 (4.8)	0.5
9	*afaCc-fimH-papC*	2 (3.3)	2 (5.2)	0	0.5
10	*aer-afaCc-fimH-papC*	13 (21.7)	4 (10.3)	9 (42.9)	0.034

## Discussion

Virulence determinants are a key factor responsible for the disease-causing ability of pathogenic strains of bacteria [2]. These virulence genes confer specific advantages to their host, and hence some of these genes may be more useful in isolates from specific sources than others. The virulence determinant *fimH*, is a gene that encodes for the FimH protein, an adhesin found in type 1 fimbriae that mediates bacterial attachment [23]. This protein has been described as a major factor in * E. coli* colonization. Similar to the results observed in this study, other studies have described a high occurrence of the *fimH* gene in *E. coli* strains from a surprisingly wide variety of sources. Hojati and colleagues reported a 92.8% occurrence in uropathogenic *Escherichia coli* (UPEC) isolates from cases of UTI. While two other studies report rates of 78.4 and 100%, respectively, also from UPEC [24,25]. Similarly, high levels (96%) were noted in avian pathogenic *Escherichia coli* strains from chicken [26] and in *E. coli* (83.3%) from a variety of non-clinical sources [27], as well as in *E. coli* (93.1%) from surface waters [28]. Considering this high occurrence of *fimH* in a wide variety of *E. coli* from different sources, it might be more advantageous to assess for specific *fimH* allelic variants as described by Qin and colleagues, as these may be more clinically relevant [29]. Another study states that the role of *fimH* is enhanced by a synergistic action with another adhesin protein [30]. The results of this study suggest that this might be the case and it is an angle worth exploring.

Similar to reports of this study, the *aer* gene has been previously reported as the second most commonly occurring among virulence genes tested [31,32]. The *aer* gene is a siderophore gene that equips the strains carrying it with the ability to acquire iron from their environment. They also help shield the strains from the toxicity of iron [33]. This might explain the higher occurrence of this gene in non-human isolates in comparison to human isolates. The level of occurrence of the gene in this study was actually similar to previous reports, which noted values ranging from 62.6% to –75% [24,33,36]. The occurrence was however much higher than the 33.3% reported by Dhaouadi and colleagues and the 47% reported by Allami and colleagues [32,37]. The Dhaouadi study only focused on a specific subset of *E. coli* and isolated various infectious conditions in cows and chickens [32]. The majority of the studies focused on human pathogens. One study offering a comparison between pathogens from different human samples observed the least occurrence of the *aer* gene from urine samples (47.8%) in comparison with blood (75.4%) and skin and soft tissue (63.1%) samples [34]. The absence of the *hlyA* gene, which has been reported as one of the most important virulence characteristics associated with the UPEC strains [33] indicates perhaps a lack of true uropathogenic isolates. Though this might be a reflection of the small sample size.

A combination of tested virulence determinants present in an organism makes up the virulence profile. Knowledge of this virulence profile of isolates is key to understanding the risk associated with specific isolates and the risk of spread from various sources. This study showed that five virulence gene profiles were unique to either human or non-human sources. Three of these were unique to isolates from human sources and two to isolates from non-human sources. The numbers of isolates with these five gene profiles were however too small (11.7 %, 7/60) to draw any conclusions. A previous study observed that there were delineations in the occurrence of virulence gene profiles in different phylogenetic groups of * E. coli* in their study [38]. Additionally, a more recent review study reported a clear prevalence of certain virulence gene profiles in pathogenic as opposed to both faecal and commensal strains [39]. This study was not carried out in Nigeria and highlights the need for more of these kinds of studies to compare data generated from this locale to that from other countries. A previous study reported that a greater than 50% occurrence of both *fimH* and *aer* in UPEC strains might perhaps point to their relevance in these isolates [24]. However, a higher occurrence of these in *E. coli* from non-human (95.2%) as opposed to human (64.1%) sources indicates this theory needs to be further explored. One key study carried out in Nigeria presented data on the occurrence of 10 virulence genes in diarrhoeagenic and non-diarrhoeagenic strains of *E. coli* from Rivers in Nigeria but failed to report on virulence gene profiles [13]. This similar lack of reporting of virulence profiles was noted in several other studies [40,43].

This study reports two predominant virulence gene profiles comprising more than 50% (55%, 33/60) of isolates (*aer-afaCc-fimH* and *aer-afaCc-fimH-papC*). This is significantly different from a 2020 study that studied just 4 virulence genes in uropathogenic *E. coli* from patients in Iran and found that the majority of isolates (47.7%) exhibited the aer-*fimH* profile [24]. These two predominant virulence gene profiles reflect the importance of adhesive factors, as they are comprised of two and three adhesive factors in association with the siderophore gene, respectively. Nuhu *et al*. (2020), assessing virulence genes in *E. coli* isolated from clinical isolates in Sokoto, Nigeria, report virulence profiles with one adhesive factor and a siderophore gene in 21.7% (5/23) of isolates, while virulence profiles with two adhesive factor and a siderophore gene were noted in 8.7% (2/23) [44]. Another study involving soil and sediment samples from duck farms in China detected similar levels of 16.7% (5/30) and 13.3% (4/30), respectively [45].

Key limitations associated with this study include the small sample size, the inability to associate faecal and urine isolates with specific disease conditions and the lack of typing of the isolates to confirm that the isolates being studied do not belong to the sample strain or clones.

## Conclusion

Results show that the *E. coli* from human and non-human sources assessed in this study do not carry distinct virulence gene profiles. Studies on a larger subset of isolates would however be necessary to determine if the virulence genes tested in this study really cannot be used to tell whether an isolate is from a human source or not in the South–South of Nigeria. Furthermore, more robust studies are however essential to check how widespread this phenomenon is, as due to the limited sample size, the isolates and hence results might not be representative of isolates in this locale.

## Abbreviation

UTI: urinary tract infection
